# Comparison of “Live High-Train Low” in Normobaric versus Hypobaric Hypoxia

**DOI:** 10.1371/journal.pone.0114418

**Published:** 2014-12-17

**Authors:** Jonas J. Saugy, Laurent Schmitt, Roberto Cejuela, Raphael Faiss, Anna Hauser, Jon P. Wehrlin, Benjamin Rudaz, Audric Delessert, Neil Robinson, Grégoire P. Millet

**Affiliations:** 1 ISSUL, Institute of Sport Sciences, Faculty of Biology and Medicine, University of Lausanne, Lausanne, Switzerland; 2 Department of Physiology, Faculty of Biology and Medicine, University of Lausanne, Lausanne, Switzerland; 3 National School of Mountain Sports/National Ski-Nordic Centre, Prémanon, France; 4 Departmental Section of Physical Education and Sports, University of Alicante, Alicante, Spain; 5 Swiss Federal Institute of Sport, Magglingen, Switzerland; 6 Swiss Laboratory for Doping Analyses, University of Lausanne, Lausanne, Switzerland; Vanderbilt University Medical Center, United States of America

## Abstract

We investigated the changes in both performance and selected physiological parameters following a Live High-Train Low (LHTL) altitude camp in either normobaric hypoxia (NH) or hypobaric hypoxia (HH) replicating current “real” practices of endurance athletes. Well-trained triathletes were split into two groups (NH, n = 14 and HH, n = 13) and completed an 18-d LHTL camp during which they trained at 1100–1200 m and resided at an altitude of 2250 m (P_i_O_2_  = 121.7±1.2 vs. 121.4±0.9 mmHg) under either NH (hypoxic chamber; F_i_O_2_ 15.8±0.8%) or HH (real altitude; barometric pressure 580±23 mmHg) conditions. Oxygen saturations (S_p_O_2_) were recorded continuously daily overnight. P_i_O_2_ and training loads were matched daily. Before (Pre-) and 1 day after (Post-) LHTL, blood samples, VO_2max_, and total haemoglobin mass (Hb_mass_) were measured. A 3-km running test was performed near sea level twice before, and 1, 7, and 21 days following LHTL. During LHTL, hypoxic exposure was lower for the NH group than for the HH group (220 vs. 300 h; P<0.001). Night S_p_O_2_ was higher (92.1±0.3 vs. 90.9±0.3%, P<0.001), and breathing frequency was lower in the NH group compared with the HH group (13.9±2.1 vs. 15.5±1.5 breath.min^−1^, P<0.05). Immediately following LHTL, similar increases in VO_2max_ (6.1±6.8 vs. 5.2±4.8%) and Hb_mass_ (2.6±1.9 vs. 3.4±2.1%) were observed in NH and HH groups, respectively, while 3-km performance was not improved. However, 21 days following the LHTL intervention, 3-km run time was significantly faster in the HH (3.3±3.6%; P<0.05) versus the NH (1.2±2.9%; ns) group. In conclusion, the greater degree of race performance enhancement by day 21 after an 18-d LHTL camp in the HH group was likely induced by a larger hypoxic dose. However, one cannot rule out other factors including differences in sleeping desaturations and breathing patterns, thus suggesting higher hypoxic stimuli in the HH group.

## Introduction

Live High - Train Low (LHTL) training camps are commonly used by athletes under either normobaric hypoxia (NH) [1]–[5] or hypobaric hypoxia (HH) [6]–[10] conditions. These two types of hypoxia are obtained by the combination of a lowered value of barometric pressure (PB) and/or a reduced inspired fraction of oxygen (F_I_O_2_) (NH: F_I_O_2_ <20.9%; PB = 760 mmHg vs. HH: F_I_O_2_ = 20.9%; PB<760 mmHg) resulting in an inspired partial pressure of oxygen (P_I_O_2_) less than 150 mmHg. NH and HH were, until recently, thought to be interchangeable since P_I_O_2_ was assumed as the only factor influencing the physiological responses to hypoxia [11]. This “*equivalent air altitude model*” [12] has now been criticized and a growing body of literature has reported physiological differences between acute exposures to NH and HH [13], [14]. Specifically, acute mountain sickness (AMS) symptoms are less severe under NH than HH conditions [15]. Pre-acclimatisation at real altitudes (HH) resulted in a significant decrease in the severity of AMS under HH conditions [16], which was not the case in individuals subjected to pre-acclimatisation under NH conditions [16]. Furthermore, according to Fulco et al., 2011, NH and HH could not “*be used interchangeably*“ and do not exhibit the same levels of effectiveness relative to pre-acclimatisation strategies for the prevention of AMS (*e*.*g*., significant decrease in the severity of AMS under HH conditions following pre-acclimatization under HH but not NH) and for the improvement of exercise performance at higher altitudes [16]. In addition, minute ventilation was lower under HH than NH conditions with the combination of lower tidal volumes and higher respiratory frequencies [17]. Interestingly, oxidative stress markers were also elevated when individuals were continuously exposed to HH conditions compared to NH for 24 h, whereas nitric oxide (NO) in exhaled air and plasma was lower under HH *versus* NH [18]. Moreover, exhaled NO and NO end-products (NO_x_) decreased in HH but remained stable in NH [18]. While the afore-mentioned studies support our recent suggestion that “*HH is a more severe environmental condition*” [14], this would also make the assumption that larger physiological adaptations would occur after prolonged hypoxic exposure under HH compared to NH conditions realistic. This still needs to be demonstrated in research.

Of interest is that the training practices of athletes are dependent upon hypoxic conditions, which reflect protocols described in the literature. It has indeed been reported that: 1) daily hypoxic exposure is shorter in NH (*i*.*e*. 8–12 h.d^−1^; [4], [5]) compared to HH (*i*.*e*. 16–18 h.d^−1^ in HH; [7], [19]) during LHTL protocols; 2) total hypoxic dose is reduced accordingly in NH (*i*.*e*. ∼150–300 h; [1], [4], [5], [20], [21]) compared to HH (*i*.*e*. 300–600 h; [9], [22]–[25]); 3) total camp duration varies between NH (11 to 23 days [2], [3], [26]; and HH (13 and 28 days; [9], [22]–[25]). Moreover, the mean performance improvements (*i*.*e*. power output increases) following LHTL were lower when the intervention was completed under NH compared to HH conditions (0.6% vs. 4.0%) [27]. Finally, most of the LHTL studies conducted under NH conditions did not elicit any performance improvement, although some induced positive erythropoietic responses [1], [28], and most of the studies reporting both performance and erythropoietic enhancements were performed under HH conditions [19], [29]. However, these findings of the differences in the physiological responses to NH vs. HH, which suggest larger adaptations in the HH condition, are based only on short-term hypoxic exposure. To the best of our knowledge, no study to date has directly compared altitude-induced adaptations (*i.e.* haematological, peripheral oxygen saturation, etc.) and performance changes following LHTL training camps under both NH and HH conditions. This is an important issue as the development of NH facilities (*e.g.*, nitrogen houses; hypoxic rooms, etc.) worldwide is increasing, and since coaches and athletes often consider NH and HH conditions to provide the same hypoxic stimulus. The issue of whether larger additional benefits occur by LTHL using HH conditions rather than NH conditions has never been directly investigated. Therefore, the present study aimed to compare the physiological responses and the performance gains in trained triathletes during and after LHTL camps with matched P_i_O_2_ in NH versus HH conditions. Of importance is that we replicated common or ‘real’ altitude training practices of endurance athletes (*e.g.*, daily exposure, total hypoxic doses under NH and HH conditions, respectively). We hypothesised that a LHTL intervention conducted under HH compared to NH should be advantageous for both physiological adaptation and performance increases.

## Methods

### Experimental Design

**Figure 1:**
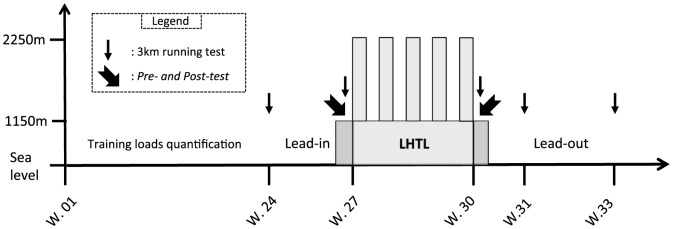
Overview of the study design separated by weeks (W.) and in order of the testing altitude, including the six months before the lead-in period where the training loads were assessed, the lead-in, the LHTL camp of 18 days, and the lead-out period. Testing included 3-km test  =  the 3-km running tests on the track near sea level; Pre-, Post-, Post-7 and Post-21  =  testing sessions; LHTL =  Live High-Train Low training camp for normobaric hypoxia (NH) and hypobaric hypoxia (HH), where athletes were exposed to both higher altitude for living and sleeping and lower altitude for training.

Our experimental design (Fig. 1) consisted of a 33-week period divided into the following four phases: 1) 24 weeks (January to May) were completed at sea level where training loads were quantified; followed by 2) a 3-wk lead-in period at sea level during which all training sessions were supervised and loads were quantified; 3) an 18-d LHTL training camp under either NH or HH conditions; and 4) a 3-wk post-altitude period at sea level during which all training sessions were once again supervised and loads were quantified.

Two groups (NH, n = 14 and HH, n = 13) were matched based on the VO_2_ max values prior to the training camp and completed an 18-d LHTL camp during which all athletes trained at 1100–1200 m and resided at an altitude of 2250 m (P_i_O_2_ = 121.7±1.2 vs. 121.4±0.9 mmHg) under either NH (hypoxic house; exposure 12.2±0.3 h.d^−1^; F_i_O_2_ 15.8±0.8%, Prémanon, France) or HH conditions (real altitude; 16.8±3.1 h.d^−1^; barometric pressure 580±23 mmHg, Fiescheralp, Switzerland). Normobaric hypoxia was obtained by extracting oxygen from ambient air (OBS, Husøysund, Norway). Calculations of F_I_O_2_ values corresponding to the required altitude took into account the altitude of Prémanon. As gas compositions were continuously monitored, O_2_ fractions were permanently adjusted during sessions in order to maintain stability. Moreover, it was determined that opening the door several times for periods of a few seconds did not change F_I_O_2_ values. For safety reasons, O_2_ and CO_2_ compositions were monitored. Each room was connected to a central monitoring station under the control of an independent investigator.

The NH group was split into smaller groups and used hypoxic chambers, whereas the HH athletes went into the valley twice daily via cable car to perform the training.

Before (Pre-) and 24 h after (Post-) the LHTL period, several physiological tests were performed on both groups in the same location (Prémanon, France, 1150 m). These sessions were conducted in a well-ventilated laboratory (temperature 22±1°C) in the same order and at the same time of the day in the Pre- and Post- testing condition. Measurements included blood samples, anthropometric measurements, maximal incremental tests on an cycle ergometer (VO_2max_), and total haemoglobin mass (Hb_mass_) assessments. Subjects performed five 3-km running tests at the following times: prior to lead-in, before LHTL, after LHTL, seven days after LHTL (Post-7), and twenty one days following LHTL (Post-21). All 3-km running tests were performed near sea level (100–390 m).

### Subjects

Twenty-seven well-trained male triathletes living at or near sea level (age 23±4 years, body height 179±5 cm, body weight 71±7 kg, fat mass 10.1±1.6%, and VO_2max_ 66.9±8.4 mL•kg^−1^•min^−1^) participated in this study. Three subjects were excluded following the lead-in period due to insufficient training loads and fitness. All subjects were non-smokers who had not been acclimatised or recently exposed to testing altitudes. Volunteers provided their written, voluntary, informed consent before participation. The experiment was approved by a Medical Ethics Committee (Commission Cantonale Valaisanne d'Ethique Médicale, CCVEM; Agreement 051/09) and performed in accordance with the Declaration of Helsinki.

## Measurements

### Training content and training loads

Two experienced certified coaches supervised and advised the athletes during all training sessions of the lead-in period and matched training content and loads for both groups during the LHTL period. Additionally, each subject's daily training loads were quantified individually by both subjective and objective means to evaluate their effects on physiological adaptation and each subject's subsequent performance. Training load quantification was performed using the ‘Objective Load Scale’ (ECOs) [30]. The training loads for the triathletes included in our study were similar to those described in other studies involving endurance athletes. The Objective Load Scale allowed for the quantification of all training loads in each sport of the triathlon (swim, bike, run, and transitions). The daily and weekly training loads (ECOs) of each subject were quantified based on each subject's physical characteristics and training program intensity. Volume was quantified by time and allowed for better comparisons of different performance levels and conditions (*e.g.*, ground surface, environmental temperature) [30].

### 3-km run test

The 3-km running tests were completed on a 400 m outdoor synthetic track near sea level. To avoid any group or pacing influences, starts were given in time-trial form (*e.g.*, 30 s between each start and randomisation of the order in which each athlete competed).

### Incremental cycling test

VO_2max_ was tested before and after LHTL using subjects' own bicycles, which were linked to a computerised ergometer system (Cyclus 2, RBM elektronik-automation GmbH, Leipzig, Germany). The exercise protocol began with a warm-up period of 5 min at a workload of 90 W. The workload was subsequently increased by 30 W•min^−1^ until voluntary exhaustion. During the final minutes of the test, subjects were strongly encouraged to perform until they reached maximal exhaustion and had achieved VO_2max_ based on the standard criteria for all tests. Each subject wore a mouthpiece and nose clip for breath collection. O_2_ and CO_2_ levels in expired gas were continuously measured and monitored as breath-by-breath values (Ultima Cardio 2 gas exchange analysis system, MGC Diagnostics with Breezesuite software, Saint Paul, MN, USA). Both the gas analyser and the flowmeter of the gas analyser were calibrated prior to each test. The highest 30 s average value served as the VO_2max_. Maximal heart rates (HR_max_) and the lowest S_p_O_2_ values were each recorded during the same time period. The maximal power output (P_max_) was the load of the last stage completed.

### Anthropometrics values

Athletes' body weights and heights were measured in the morning before breakfast.

### Haemoglobin mass

Hb_mass_ was measured in duplicate by using a slightly modified version [31] of the optimised carbon monoxide (CO)-rebreathing method described by Schmidt and Prommer [32]. The subjects inhaled a bolus of 100 mL of pure CO (Multigas SA, Domdidier, Switzerland), followed by 3.5 L of oxygen. Each Hb_mass_ measurement was performed in duplicate on two consecutive days (12- to 24-h time lag between measurements). Across all time points, the mean error for duplicate Hb_mass_ measurement was 2.1% in our mobile laboratory. Hb_mass_ data are expressed as the mean values of the duplicate measurements.

Values of red cell volume (RCV), blood volume (BV), and plasma volume (PV) were estimated using the following formulas: RCV  =  Hb_mass_/MCHC ×100, BV  =  RCV × (100/Hct) and PV  =  BV – RCV, where MCHC is the mean corpuscular haemoglobin concentration, and Hct is the haematocrit corrected to whole-body haematocrit by the cell factor of 0.91. For the calculation of RCV, BV, and PV, venous haemoglobin concentrations [Hb] and venous Hct were used. Blood gas analyses were conducted using an ABL 800flex (Radiometer A/S, Copenhagen, Denmark).

### Blood samples

Antecubital vein blood samples (4.9 mL EDTA tube, Sarstedt, Nümbrecht, Germany) were taken during three time periods including either before breakfast or immediately after waking up, before LHTL, and after LHTL. Blood was subsequently analysed via fluorescent flow cytometry and hydrodynamic focusing (XT-2000i, Sysmex Europe, Norderstedt, Germany), and the following primary haematological parameters were quantified: red blood cells (RBC), haemoglobin (Hb), haematocrit (Hct), mean cell volume (MCV), mean cell haemoglobin (MCH), mean cell haemoglobin concentration (MCHC), reticulocyte percentage (RET%), absolute number of reticulocytes (RET#), and immature reticulocyte fraction (IRF). The Sysmex XT-2000i underwent regular internal quality control procedures as required by the standards of laboratory medicine. During the period of our study, the coefficient of variations (CV), which was determined using internal quality controls, was far below 1.5% for Hb and 1.5% for RET% (within CV limits accepted by the manufacturer of the instrument). Plasma EPO was quantified using a standard procedure with an ELISA kit (Stemcell Technologies, Grenoble, France). Plasma EPO concentrations below the limit of quantification (1.6 mU/mL) were excluded from the analyses. CVs determined by three internal quality controls (levels: low, medium and high) were below 15% in our WADA (World Anti-Doping Agency) accredited laboratory [33]. All plasma samples were analysed in duplicate, where the mean values of the duplicate were used for this study. Additionally, baseline ferritin was quantified using standard laboratory procedures (Dimension EXL, Siemens Healthcare Diagnostics SA, Zürich, Switzerland) to evaluate the subject's iron stores. It is important to note that all athletes were tested for doping by the accredited laboratory according to the standards of the biological passport. This was done to avoid performance enhancement via doping.

### Questionnaires

Subjects completed three different questionnaires on a daily basis immediately after waking up (hypoxic rooms for NH and normal rooms for HH, but all were hypoxic) and during three phases before, during, and after the LHTL training camp. The three questionnaires were as follows: 1) *The Lake Louise score questionnaire, 2) The Daily Analysis of Life Demands for Athletes (DALDA), and 3) Sleep assessment questionnaire.*

The Lake Louise score questionnaire is scoring system developed by the 1991 International Hypoxia Symposium consensus committee, which met at Lake Louise in Alberta, Canada. It is widely used today to assess the severity of AMS. The DALDA is a self-reported sport-specific tool describing the stress sources and characteristics of each person, which allows for the differentiation of the individuality of stress responses. This questionnaire is divided into two parts; Part A describes the general stress sources that occur in everyday life for an athlete (diet, home life, school, work, friends, training, climate, sleep, recreation, and health); and Part B determines which symptoms of any existence in stress reactions of the athlete. The sleep assessment questionnaire was the Groningen Sleep Quality Scale (GSQS), which was used to evaluate for high altitude sleep (HAS) disturbances. It consists of a sleep quality score (GSQSS) and two visual analog scales (VAS), which yield a score between 0 and 10 for sleep quality and waking state.

### Sleep assessment

S_p_O_2_ and HR were recorded each evening at 0.25 Hz with a wrist oximeter connected to a finger sensor (Wristox 3150 with 8000SM-WO Sensor, Nonin, Plymouth, MN). Subjects wore an instrumented t-shirt (model SEW, CSEM, Neuchâtel, Switzerland) each night (including the 2 nights before and the 2 nights after LHTL), a device made of comfortable fabric that was used to measure breathing frequency via an elastic sensor included in the textile, as well as each subject's sleeping position via accelerometers.

### Data Analysis and Statistics

Data are reported as the means and standard deviations. Data were tested for equality of variance (Fisher-Snedecor *F-test*) and for normality (Shapiro-Wilk test). When both conditions were met, a two-way ANOVA was performed for repeated measures for each condition (NH and HH) to determine time effects for variables measured on several occasions during the camps with pairwise multiple comparison procedures (Holm-Sidak method). Differences between results obtained before and after LHTL for both the NH and HH groups were subsequently also compared using a two-way ANOVA. Differences in percentage changes between the groups were tested with a Wilcoxon signed rank sum test. When either equality of variance or normality were not satisfied, variables were analysed for each condition using a Friedman test for repeated measures to determine time effects using pairwise multiple comparison procedures (Bonferroni test). In this case, differences between the NH and HH groups at baseline (Pre-) were tested using a Mann-Whitney rank sum test. The correlation between values of Hb_mass_ initial (in g) or pre-to-post change (in %) and VO_2max_ (mL.kg^−1^.min^−1^) as well as correlations between all haematological and physiological parameters were calculated via the Pearson product moment correlation. Null hypotheses were rejected at *P*<0.05. All analyses were completed using Sigmaplot 11.0 software (Systat Software, San Jose, CA).

## Results

### Training loads

**Figure 2:**
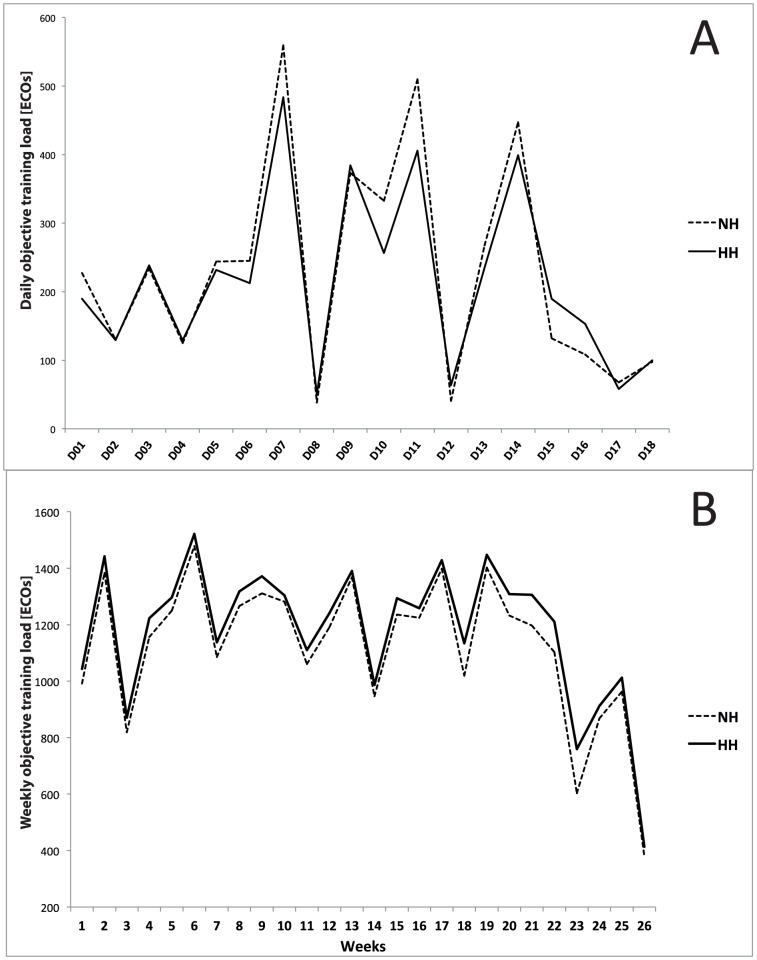
A. Daily objective training (D01-D18: day 01 to day 18) loads during the Live High-Train Low (LHTL) camp for normobaric hypoxia (NH) and hypobaric hypoxia (HH) groups. **B**. Weekly objective training loads during the six months before the intervention for NH and HH groups.

No differences were found in daily or average training loads between the two groups during the 18-d LHTL camp (232±159 vs. 217±129 ECOs for the NH and HH groups, respectively; Fig. 2A). Additionally, no differences were found in weekly training loads monitored during the 6 months prior to the study (1161±130 vs. 1208±168 ECOs per week for the NH and HH groups, respectively; Fig. 2B), nor were any differences noted during the lead-in period or the post-hypoxic period.

### 3-km performance test

**Figure 3:**
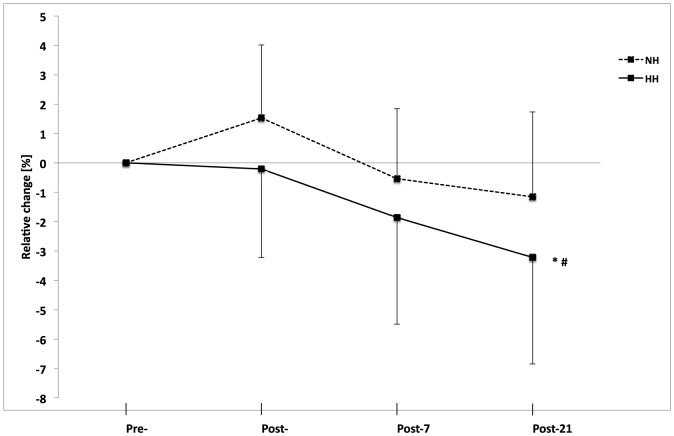
Relative change in 3-km run time from Pre- to Post-, Post-7, and Post-21 as determined on a running track near sea level for the normobaric hypoxia (NH) and hypobaric hypoxia (HH) groups (in %). Data are mean±standard error *P<0.05 for differences with Pre- and ^#^P<0.05 for differences between groups.

Compared to Pre-, 3-km performance remained unchanged at Post- and Post-7 in both groups. Whereas run performance did not improve significantly in the NH group (630.1±64.8 vs. 621.8±54.8 s, P>0.05) from Pre- to Post-21, faster 3-km run times occurred in the HH group (611.1±48.5 vs. 588.3±32.2 s; −3.3±3.6%, P<0.05, Fig. 3). No differences were found between groups during the lead-in period, before LHTL, after LHTL, or 7 days post-LHTL. In addition, no differences were found between Lead-in and Pre- for both groups (626.3±63.8 vs. 630.1±64.8 s and 602.4±44.3 vs. 611.1±48.5 s, for NH and HH at Lead-in vs. Pre-, respectively).

### Maximal test on cycle ergometer

These results are presented in Table 1. Both groups increased their maximal oxygen uptake values and power output values by the same amount immediately after the LHTL training camp period (+6.1±6.8 vs. +5.2±4.8% VO_2max_ and +9.6±5.2 vs. +6.6±4.7% P_max_, for the NH and HH groups, respectively).

**Table 1:** Physiological parameters before (Pre-) and after (Post-) the Live High-Train Low (LHTL) camps for the normobaric hypoxia (NH) and hypobaric hypoxia (HH) groups.

		Pre-	Post-	Delta %
VO_2max_	NH	65.4±7.8	69.1±5.6 **	6.1±6.8
[mL.•kg^−1^•.min^−1^]	HH	69.2±8.9	73.2±7.1 **	5.2±4.8
HR_max_	NH	187±7	188±5	0.6±2.6
[b•.min^−1^]	HH	185±9	185±8	0.1±1.9
P_max_	NH	353±43	385±38 ***	9.6±5.2
[W]	HH	378±24	403±32 ***	6.6±4.7
VE_max_	NH	178.9±17.8	184.3±14.6	3.7±10.2
[l•.min^−1^]	HH	182.6±34	188.1±19.3	4.5±9.3

VO_2max_ maximal oxygen uptake; HR_max_ maximal heart rate; P_max_ maximal power output; VE_max_ maximal ventilation. Data are mean ± SD; **P<0.01 and ***P<0.001 for differences between Pre- and Post-.

### Body fat mass and weight

Body weight (69.5±5.9 vs. 69.6±5.6 kg for the NH group and 69.9±6.4 vs. 69.1±6.2 kg for the HH group) and fat mass percentage (9.9±1.8 vs. 9.1±1.3% for the NH group and 10.3±1.4 vs. 8.4±0.7% for the HH group) did not differ between groups.

### Night S_p_O_2_ and heart rate

**Figure 4:**
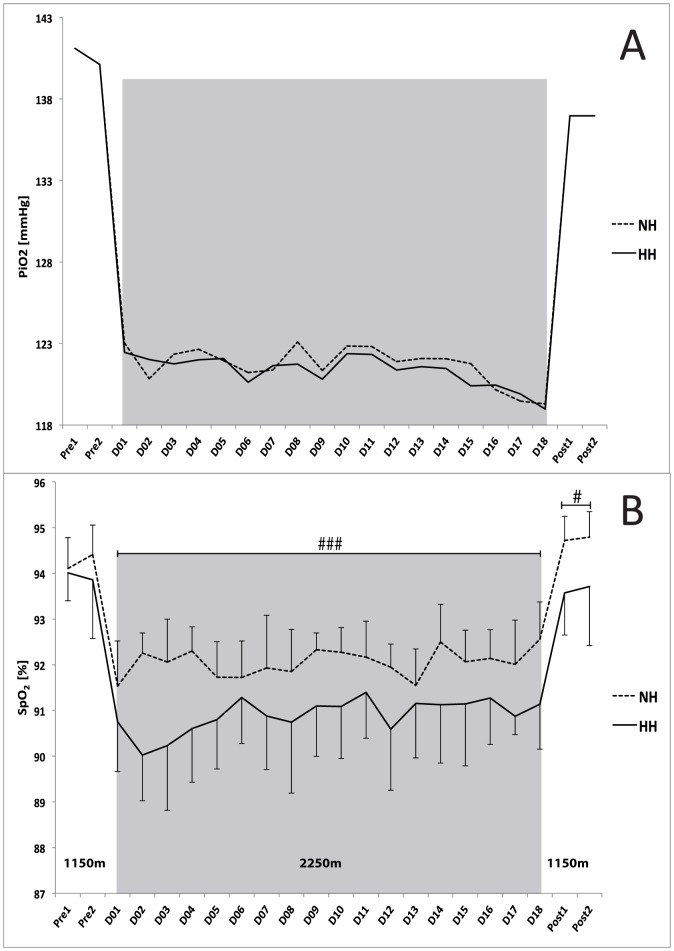
A. Daily values of inspired pressure of oxygen (P_i_O_2_ in mmHg) during the Live High-Train Low (LHTL) camps for the normobaric hypoxia (NH) and hypobaric hypoxia (HH) groups. **B**. Mean values of night oxygen pulse saturation (S_p_O_2_). Data are presented in mean ± standard error. Pre1-Pre2: measurements before the camps (1150 m, Prémanon, France); D01-D18: measurement during the camps (NH: hypoxic room in Prémanon, France; HH: Fiescheralp, Switzerland). ^#^P<0.05, ^###^P<0.001 for differences between groups.

No differences in average values of night HR were found between the groups or between different days (51±1 and 50±2 bpm, for the NH and HH groups, respectively). Conversely, although mean S_p_O_2_ values (Fig. 4B) were similar during the control nights (before the camps, Pre1 and Pre2), they were higher in the NH group than in the HH group between day 1 and day 18 (D1 to D18) (92.1±0.3 vs. 90.9±0.3, for the NH and HH groups, respectively; P<0.001) and remained higher (P<0.05) during each of the two nights following the camps (Post1: 94.7±0.5 vs. 93.5±0.9% and Post2: 94.8±0.6 vs. 93.7±1.3%).

### Breathing frequency

The average values of breathing frequency were similar between the groups during the two nights prior to the camp (14.0±1.8 and 13.9±1.5 breath.min^−1^ for NH and HH, respectively). However, breathing frequencies were lower in the NH group than in the HH group (13.9±2.1 vs. 15.5±1.5 breath•min^−1^, P<0.05) during the LHTL camp (D1 to D18) and remained lower upon the camp's completion (13.7±1.9 vs. 15.1±1.3 breath•min^−1^, P<0.05).

### Total Haemoglobin mass

All results are presented in Table 2. Both groups increased their total haemoglobin masses during the study period (912±96 vs. 936±103 g and 950±115 vs. 967±122 g for the NH and HH groups, respectively, P<0.001).

**Table 2:** Haematological parameters before (Pre-) and after (Post-) the Live High-Train Low (LHTL) camps for the normobaric hypoxia (NH) and hypobaric hypoxia (HH) groups.

		Pre-	Post-	Delta %
EPO	NH	3.85±1.42	3.37±1.59	−14.1±18.3
[mU/mL]	HH	4.96±3.73	3.92±3.94*	−34.5±27.5^#^
RBC	NH	5.26±0.39	5.08±0.46	−3.5±4.8
[u/µl]	HH	5.15±0.36	5.18±0.43^##^	0.6±3.8^#^
HGB	NH	15.75±1.07	15.75±1.14	0.1±5.1
[g/dl]	HH	15.53±0.88	16.18±1.08*^#^	4.2±3.9^#^
Hct	NH	46.25±2.89	45.19±2.97	−2.2±5.6
[%]	HH	45.24±2.43	46.33±2.62*^#^	2.5±3.8^#^
MCV	NH	88.06±3.71	89.22±3.16 **	1.4±1.3
[fl]	HH	88.06±4.74	89.69±4.51 ***	1.9±1.4
MCH	NH	29.96±1.13	31.09±1.27 ***	3.8±0.9
[pg]	HH	30.22±1.27	31.32±1.34 ***	3.6±1.2
MCHC	NH	34.03±0.72	34.85±0.58 ***	2.4±1.2
[g/dl]	HH	34.33±1.01	34.92±0.79 ***	1.7±2.0
RET	NH	0.89±0.31	1.03±0.28 **	21.6±30.0
[%]	HH	0.98±0.24	1.18±0.38**^#^	23.2±34.1^#^
IRF	NH	6.72±3.61	5.37±2.17	−9.02±33.95
[%]	HH	6.06±1.77	4.76±2.75	−19.09±36.46
Hb_mass_	NH	912.4±96.6	935.9±102.6 ***	2.6±1.9
[g]	HH	946.8±126.7	978.6±131.6 ***	3.4±2.1
RCV	NH	2675.8±295.4	2692.1±289.9	0.7±2.8
[ml]	HH	2734.1±306.5	2778.7±324.1	1.64±3.1
BV	NH	6358.3±583.8	6553.6±664.1	3.1±4.8
[ml]	HH	6617.1±744.1	6557.5±821.4	−1.0±4.2^##^
PV	NH	3682.6±384.6	3861.6±460.9	5.1±8.6
[ml]	HH	3883.1±505.3	3778.8±551.8	−2.7±6.1^##^

EPO erythropoietin; RBC red blood cells; HGB haemoglobin; Htc hematocrit; MCV mean cell volume; MCH mean cell haemoglobin; MCHC mean cell haemoglobin concentration; RET reticulocytes; IRF immature reticulocyte fraction; Hb_mass_ haemoglobin mass; RCV red cell volume; BV blood volume; PV plasma volume. Data are mean ± SD; *P<0.05, **P<0.01 and ***P<0.001 for differences between Pre- and Post-; ^#^P<0.05 and ^##^P<0.01 for differences between NH and HH.

### Blood Parameters

All blood parameters are presented in Table 2. The RBC number, [Hb] and Hct were each lower in the NH group than in the HH group following camp. Additionally, the initial ferritin values were not different between groups and were within reference ranges (98.7±75.9 *vs*. 105.3±51.9 ng/mL for NH and HH, respectively). A larger decrease in [EPO] was noted in the HH group compared with the NH group with return to 1150 m (table 2).

### Hypoxic doses and P_i_O_2_

The daily (12.2±0.3 vs. 16.8±3.1 h, P<0.001) and total (220.1±0.9 *vs*. 302.9±5.5 h, P<0.001) hypoxic doses were lower in the NH group than in the HH group. No differences were found in either daily or average P_i_O_2_ values between the two training camps (121.7±1.2 vs. 121.4±0.9 mmHg for the NH and HH groups, respectively, Fig. 4A).

### Questionnaires

The mean Lake Louise Score was 1.2±0.4 for the NH group and 1.1±0.4 for the HH group. No differences were found between the groups. DALDA Part B results were not different between the groups, and scores did not change across days (2.3±0.7 vs. 2.3±0.6 for the NH and HH groups, respectively). DALDA Part A results included a significantly higher score for the NH group than for the HH group from D07 to D11 and D15 to D16 (P<0.05). The average VAS value for the sleep quality of the entire camp was lower in the NH group (6.0±0.4 vs. 6.4±0.4 for the NH and HH groups, respectively; P<0.001). The GSQSS was significantly higher for the NH group (4.7±1.1 vs. 3.6±0.8 for the NH and HH groups, respectively; P<0.001), indicating poorer sleep quality for the NH group than for the HH group. However, waking state VAS scores were not different between the groups (5.9±0.5 vs. 5.7±0.5 for the NH and HH groups, respectively).

### Correlations

A positive correlation was found between the mean Hb_mass_ (in g) and VO_2max_ (mL.kg^−1^.min^−1^) values of both groups following camp (r = 0.68, P<0.01 and r = 0.86, P<0.001 for the NH and HH groups, respectively). We did not find any correlations between changes in Hb_mass_ and VO_2max_ or between initial value of Hb_mass_ and any other parameter.

## Discussion

The present study demonstrated that an 18-d LHTL altitude camp performed under either NH or HH conditions induced different physiological and performance responses: (i) during LHTL, longer hypoxic exposure, larger night desaturation levels, and higher breathing frequencies were noted in the HH group; (ii) immediately after LHTL, larger haematological changes occurred in the HH group, but similar increases were observed in Hb_mass_ and VO_2max_; (iii) finally, larger performance enhancements were measured in the HH group 3 weeks after return to sea level.

Differences in daily responses were found between our experimental groups. The total hypoxic dose was higher for the HH group than for the NH group (300 vs. 220 h). The present study aimed to compare two LHTL altitude training camps (simulated versus real altitude) in “real conditions”, corresponding to those encountered by elite athletes in their training practices. In this context, the daily exposures reported in the present study (12 vs. 17 h•d^−1^ in the NH and HH groups, respectively) are consistent with those reported previously with hypoxic exposures of 8–12 h•d^−1^ [4], [5] and 18 h•d^−1^ [7], [19] in NH and HH conditions, respectively. Meta-analysis results suggest that optimal durations are of 11 and 18 h•d^−1^ [27]. There is a clear dose-response effect between the hypoxic dose and the haematological responses, as highlighted by Levine and Stray-Gundersen's study [23]. However, differences in hypoxic doses between the NH and HH groups cannot be easily reduced, as long duration confinement for individuals of the NH group may cause other complications, including detrimental reductions in plasma volume [34]. Aside from the difference in total hypoxic exposure, the HH group also experienced fewer transitions between “high” (2250 m) and “low” altitudes. LHTL under NH conditions implies numerous daily exposures to normoxia and many transitions to and from hypoxic conditions. Therefore, the exposure is more intermittent than it would be for LHTL under HH conditions (*e.g.*, the number of daily shifts between altitudes was 7 vs. 2 in the NH and HH groups, respectively). Taken together, we assume that these intermittent characteristics cannot be ruled out as explanations for noted altitude training adaptations. In addition, Navarette-Opazo & Mitchell [35] demonstrated in a recent meta-analysis, that the number of cycles per day was one of the most important variables for the efficiency of the intermittent hypoxic methods. Interestingly, in a similar way, Garvican et al. [22] described the “*oscillating nature of LHTL*,” the daily descents to sea level and the associated normoxic exposures as an explanation for the less dramatic fall in [EPO] from Pre- to Post-, compared to changes associated with continuous exposure to high altitudes. This observation may also explain the differences (almost twice as much in the HH group) in [EPO] decreases noted between the two groups.

Night measurements also indicated that the two conditions were not similar. Night arterial oxygen saturations were higher in the NH group than in the HH group, while breathing frequencies were lower (Fig. 4B). Similarly, Savourey et al. [17] first demonstrated that HH induced greater respiratory frequencies, lower tidal volumes, and minute ventilation values over short time periods; thus, suggesting higher amounts of alveolar physiologic dead space, which is associated with ventilatory alkalosis and hypocapnia [14]. Later, similar conclusions have also been reported by Richard and Koehle [13] and Faiss et al. [18]. Changes in fluid balance have also been shown with differences between the HH and NH conditions [15]. Additionally, barometric pressure (PB) modifies fluid circulation and trans-alveoli-capillary membrane flux [36]. This may induce a stronger pulmonary vasoconstriction in the HH group and modify oxygen diffusion by decreasing the pressure gradient [14]. PB may also influence N_2_ and O_2_ concentrations in cerebrospinal fluid, as well as central regulation of ventilation [12]. In addition to the reported alteration in ventilatory pattern, all these alterations might contribute also to the lower mean sleeping S_p_O_2_ values in the HH group.

It is interesting to note that these lower S_p_O_2_ values were maintained after subjects returned to 1150 m during the two nights immediately following the camp. Several studies [17], [37] have reported a more rapid blood desaturation under HH conditions, leading to a longer duration of hypoxemia. This is in line with the present results as we observed a larger decrease in S_p_O_2_ from the first night under HH conditions and the maintenance of these lower values during the whole LHTL camp. Interestingly, the higher breathing frequencies while sleeping in HH conditions at 2250 m were also maintained during the two nights spent at 1150 m following the camp. This larger desaturation, which was most likely influenced by a lower tidal volume, potentially induced more severe hypoxemia in the HH group and delayed performance enhancement.

Specific short-term post-hypoxic responses were observed immediately following the camps. These responses were noted primarily among haematological parameters and revealed differences between the groups that were clearly influenced by differences in both daily and total hypoxic exposures. To date, there is consensus on neither the dose-response relationship between hypoxic stimulus and Hbmass increase nor on the recommendation in terms of total hypoxic exposure duration. For instance, Richalet and Gore have recommended an exposure of 216 h [20], Garvican et al. of 300 h [22], and Wilber at al. a minimum of 4 weeks with at least 22 h.d^−1^ [25]. However, although the increase in Hb_mass_ immediately following camp was significant, the magnitude of the increase was the same in both groups (2.6 vs. 3.4% for the NH and HH groups, respectively). Our results are consistent with those of previous studies in which Hb_mass_ was increased by 3–4% following several LHTL protocols [7], [19], [22], [28], [38] and illustrate an enhanced oxygen transport capacity as a result of an erythropoietic response. These findings are also consistent with the dose-response relationship and correspond to an average rise of 1% per 100 h of exposure [1], [38]. Gore et al. [38] also suggested that the amount of the hypoxic dose or the level of altitude should be higher in cases involving the use of simulated altitudes to produce equivalent results at real altitudes (*i.e.* 3000 m in NH with LHTL corresponding to 2320 m in HH with classical altitude training). Further, our results showed a 5–6% increase in VO_2max_ in both groups following LHTL, an increase that is commonly observed under HH conditions [19] as well as NH conditions [26], [39], although the phenomenon is more common in the former [3], [40]. In the present study, the difference of 80 h (220 vs. 300 h for the NH and HH groups, respectively) is likely the primary cause of the larger increases in Hct, [Hb] and RET in the HH group. These larger increases with no difference in Hb_mass_ increase could also be explained by plasma shift differences.

We also reported differences in plasma and blood volume changes during the study period between the NH and HH groups. Of interest, is that diuresis and changes in fluid balance have been shown to be different between HH and NH (*i.e.* larger diuresis for NH and larger fluid retention for HH) [15], [41]. The influence of PV changes (*e.g.*, expansion and reduction) on performance enhancement are well-known [42]. It is known that plasma volume may increase until at least 16 days following an altitude training camp [43]. One may speculate that the non-significant difference in plasma volume observed at Post- between the two groups would not occur anymore at Post-21, suggesting a potential larger increase in PV for the HH group during these 3 weeks. This potential hemodynamic enhancement would partially explain the longer delay in performance enhancement compared to the NH group. However, based on the existing contradictory literature, it is difficult to speculate on the maintenance of the Hb_mass_ gains at Post-21. Garvican-Lewis et al. [44] reported a 4% increase two weeks after 11 days of LHTL under NH conditions at 3000 m (14 h.d-1). However, a persistent increase in Hb_mass_ post-exposure does not mean that it did not decrease from the initial elevation during the days following the exposure. Several studies show a relatively linear decrease in Hb and Hct starting as soon as hypoxic stimulus is removed. For example, Gough et al. [45], reported a drop from post to post-14; and Garvican et al. [22], demonstrated that Hb_mass_ had started to drop off by Post-4 and was no different from control after 10 days at sea level. Similarly, elite altitude-native Kenyan runners showed a significant 20 g decrease after 21 days at sea level (Fig. 2A in [46]).

One of the most important findings of the present study is the performance enhancement noted in the HH group three weeks after camp. Our results are consistent with those of the meta-analysis by Bonetti and Hopkins [27], which described a “terrestrial” LHTL protocol that induced additional benefits relative to the performances of elite athletes as estimated by power output increases of 4.0% under HH conditions and 0.6% under NH conditions.

Delayed (in our case, three weeks after LHTL) performance enhancement has been observed in several [7], [26] but not all studies [39]. The following mechanisms have been proposed: enhanced stroke volume compensating for the reduction in heart rate [47], enhanced efficiency [41], and increased VO_2_ and power output at the lactic threshold [26]. Recently, Chapman et al. [48] described the following three components, which may influence performance changes following altitude training: the timing of the decay of red cell mass, the consequences of ventilatory acclimatisation, and the alterations in the biomechanical and neuromuscular factors associated with force production. Regarding the first component, we cannot determine if the unknown decay of Hb_mass_ (see above) in the present study influenced the difference in performance enhancement between groups at Post-21. The observed differences in the ventilatory pattern, as evidenced by the higher night breathing frequency in the HH group, could have influenced the delayed performance difference between groups. Finally, it is unlikely that there was any biomechanical alteration in either group who trained “low” at 1100–1200 m, as suggested by the preliminary results of Laymon et al. [49]. Therefore, a hypoxic-induced alteration in running style was probably not involved in the observed difference at Post-21.

### Strengths and limitations

Our primary aim was to compare LHTL training camps under real and simulated altitude in an ecological setting (*e.g.*, by reproducing “real life” conditions of daily exposures and camp durations as described in previous LHTL studies under NH and HH conditions, respectively) rather than investigating the efficiency of LHTL, which has previously been documented. For this reason, we did not include a sea level control group. To the best of our knowledge, our study is the first to report differences in performance enhancement following direct comparisons of prolonged altitude training under NH and HH conditions. The athletes were well trained as shown by their training loads, VO_2max_, and performance levels. The groups were matched according to the VO_2max_ values. Additionally, the athletes' training load and content were quantified and matched during the 6-month period before the study, which included a suitable lead-in period. To our knowledge, this study is the first where training loads and altitude levels were entirely matched on a daily basis during the entire LHTL period. The current study emphasizes the importance of well-controlled studies to achieve a better understanding of the mechanisms and potential benefits of altitude training [50].

The primary limitation of this study was that no measurements of total haemoglobin mass or other haematological parameters were completed at one or three weeks following LHTL due to logistical constraints. In addition, since our aim was to compare two typical 18-d LHTL camps in “real” conditions, the hypoxic doses were different. One cannot rule out that the physiological and performance responses would be less dissimilar between NH and HH with a close matching of hypoxic doses.

### Perspectives

This study questions the relationship between modes of prolonged hypoxic exposure and subsequent performance improvement. Real altitude conditions (HH) were more demanding than the simulated altitude (NH) utilised in training camps of the same duration. However, in general, hypoxic chambers make adjustments possible and continue to attract interest because of their practicality. Chapman [51] emphasises that the response to training and competition at high altitudes is individual, and that timing the return to competition after altitude training must also be individualised to obtain optimal sea level performance [48]. Normobaric hypoxia devices offer these individualisation possibilities in terms of hypoxic doses and altitude adjustments. Finally, further studies are necessary to assess the physiological responses of these hypoxic training methods to equivalent hypoxic doses.

This study highlights the different physiological adaptations noted in the HH and NH LHTL camps. Our results suggest that future investigations should increase the altitude of the normobaric hypoxia group to reach the same level of desaturation as that experienced under hypobaric hypoxic conditions and lengthen the durations of the camps to obtain hypoxic doses similar to those experienced under hypobaric hypoxic conditions.

### Conclusion

The primary finding of the study is that there were significant differences in the responses to a LHTL training camp in NH compared to HH. Specifically, our results included greater performance enhancements in the HH group three weeks after LHTL, greater significance in haematological changes within the HH group following camp, greater night desaturation levels, and higher breathing frequencies in the HH group, with similar increases in Hb_mass_ and VO_2max_ following LHTL in both NH and HH. Additionally, one cannot rule out other factors, including differences in sleep quality, desaturation level, breathing patterns, fewer transitions between high and low altitudes (*e.g.*, intermittence) or different responses relative to plasma volumes and [EPO] following camp.
