# Correction: Comparison of “Live High-Train Low” in Normobaric versus Hypobaric Hypoxia

**DOI:** 10.1371/journal.pone.0133091

**Published:** 2015-07-14

**Authors:** Jonas J. Saugy, Laurent Schmitt, Roberto Cejuela, Raphael Faiss, Anna Hauser, Jon P. Wehrlin, Benjamin Rudaz, Audric Delessert, Neil Robinson, Grégoire P. Millet

The inspired fraction of oxygen (F_i_O_2_) for the normobaric hypoxic condition was incorrectly reported to be 15.8 ± 0.8%. The correct F_i_O_2_ value for the NH condition is 17.9 ± 0.2% and the correct inspired pressure of oxygen (P_i_O_2_) values were 111.1 ± 1.1 vs 111.5 ± 1.0 mmHg in normobaric and hypobaric hypoxia, respectively. The authors wish to clarify and confirm that the simulated altitude was perfectly matched to the real altitude of 2250m. The error appears only in the paper. This correction has no impact on the living altitudes between the two hypoxic conditions, as P_i_O_2_ was strictly matched between conditions.

As a result, there are errors in Fig 4. Please see the updated Fig 4 here.

**Fig 4:**
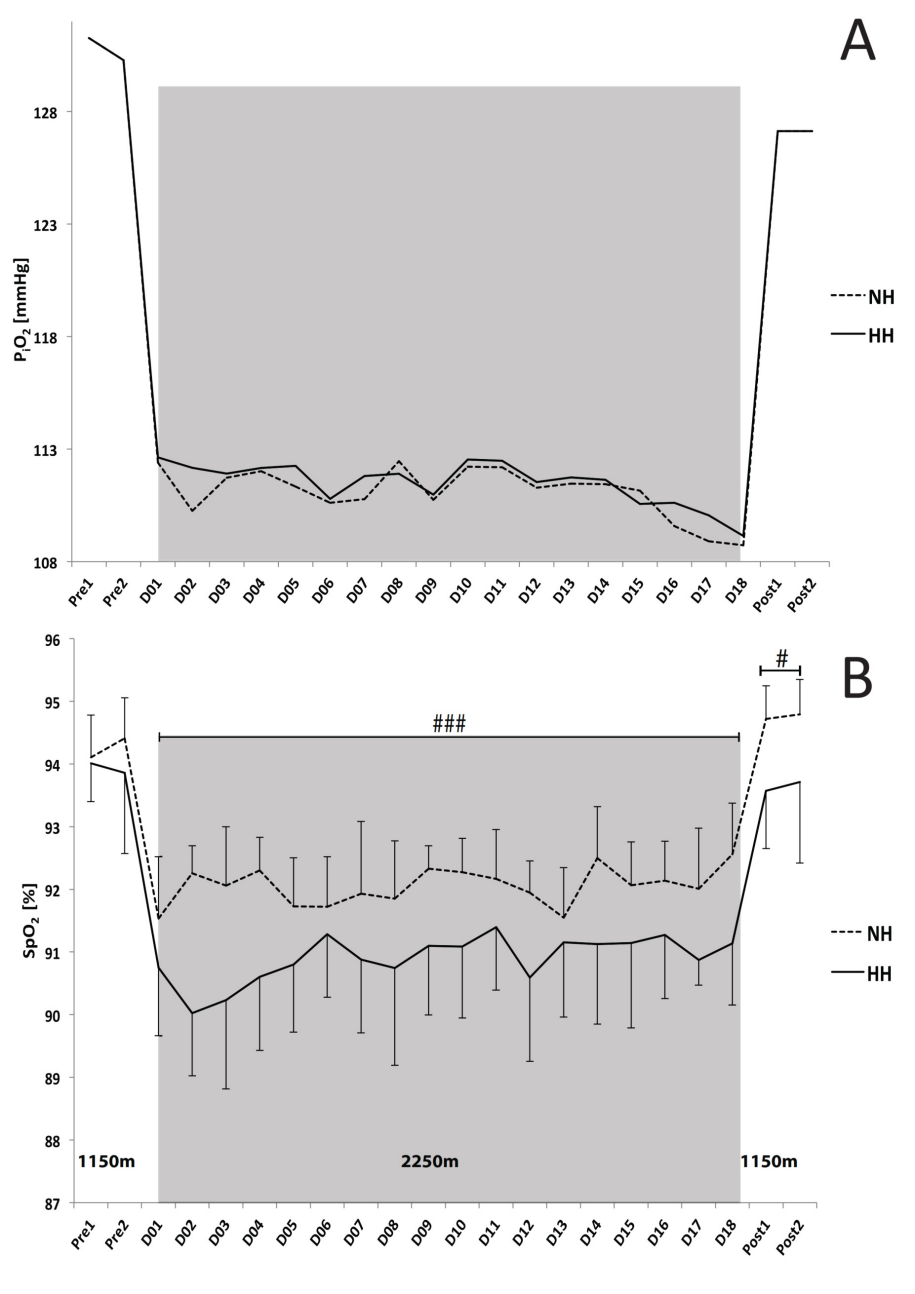
**A**. Daily values of inspired pressure of oxygen (P_i_O_2_ in mmHg) during the Live High-Train Low (LHTL) camps for the normobaric hypoxia (NH) and hypobaric hypoxia (HH) groups. **B**. Mean values of night oxygen pulse saturation (S_p_O_2_). Data are presented in mean ± standard error. Pre1-Pre2: measurements before the camps (1150 m, Prémanon, France); D01–D18: measurement during the camps (NH: hypoxic room in Prémanon, France; HH: Fiescheralp, Switzerland). ^#^P<0.05, ^###^P<0.001 for differences between groups.
